# A novel interspecies recombinant enterovirus (Enterovirus A120) isolated from a case of acute flaccid paralysis in China

**DOI:** 10.1080/22221751.2020.1796527

**Published:** 2020-07-31

**Authors:** Huanhuan Lu, Mei Hong, Yong Zhang, Jinbo Xiao, Man Zhang, Keyi Zhang, Yang Song, Zhenzhi Han, Qian Yang, Dongyan Wang, Dongmei Yan, Shuangli Zhu, Wenbo Xu

**Affiliations:** a WHO WPRO Regional Polio Reference Laboratory, National Laboratory for Poliomyelitis and National Health Commission Key Laboratory for Biosafety, National Institute for Viral Disease Control and Prevention, Chinese Center for Disease Control and Prevention, Beijing, People’s Republic of China; b Tibet Center for Disease Control and Prevention, Lhasa City, People’s Republic of China; c Center for Biosafety Mega-Science, Chinese Academy of Sciences, Beijing, People’s Republic of China

**Keywords:** EV-A120, genomic characterization, phylogenetic analysis, recombinant analysis, seroepidemiology, temperature sensitivity

## Abstract

EV-A120 is a recently identified serotype of the enterovirus A species. Only one full-length genomic sequence is currently available in GenBank, and very few studies have been conducted on EV-A120 globally. Thus, additional information and research on EV-A120 are needed to explore its genetic characteristics, phylogeny, and relationship with enteroviral disease. In this study, we report the phylogenetic characteristics of a EV-A120 strain (*Q0082*/XZ/CHN/2000) from Tibet, China. The amino acid sequence similarity and nucleotide sequence similarity of the full-length genomic sequence of this EV-A120 strain and the EV-A120 prototype strain were 96.3% and 79.9%, respectively, showing an evolutionary trend. Recombination analysis found intraspecies recombination in the *5′ -UTR*, *2B*, *2C*, and *3D* regions. Serum neutralization testing of the EV-A120 (*Q0082*) strain was also carried out. Low serum-positive rates and geometric mean titres (GMTs) indicated that the extent of EV-A120 transmission and exposure in the population was very limited compared with that in the outbreaks of EV-A71 and CV-A16 in China since 2008. The EV-A120 strain (*Q0082*) is non-temperature sensitive, indicating its potential to spread in the population. In summary, this study reports the full-length genomic sequence of EV-A120 and provides important information for its global molecular epidemiology.

## Introduction

The genus *Enterovirus*, within family *Picornaviridea*, order *Picornavirales*, contains 15 species: Enterovirus A-L, and Rhinovirus A-C. Four main species of enterovirus cause human diseases: Enterovirus A (EV-A), Enterovirus B (EV-B), Enterovirus C (EV-C), and Enterovirus D (EV-D), and these include over 100 serotypes (poliovirus, coxsackievirus A, coxsackievirus B, echovirus, and novel enterovirus) [1–3]. Human enteroviruses are small, non-enveloped, single-stranded, and positive-sense RNA viruses. Generally, enterovirus genomes contain approximately 7400–7500 nucleotides (nt), covalently attached at the 5′ end to a virus-coding protein (VPg) containing 22 amino acids. The viral RNA contains a long open reading framework (ORF) encoding approximately 2200 amino acids between the 5′-non-coding regions (*5*′*-UTR*) and 3′-non-coding regions (*3*′*-UTR*), which can be segmented into three polyprotein precursors, further divided into four structural proteins, *VP4*, *VP2*, *VP3,* and *VP1*, and seven non-structural proteins, *2A*-*2C* and *3A*-*3D* [4–6]. The *5*′*-UTR* of picornaviruses contains two main types of internal ribosome entry sites (IRES), namely type I IRES (as in poliovirus and rhinovirus) and type II IRES (present in pericardial and foot-and-mouth disease viruses). Although the sequence consistency between different members with type I IRES may be less than 50%, their secondary structure predictions are very similar [7,8].

Most enterovirus infections are asymptomatic, but in disease state, they induce a range of different clinical syndromes such as hand, foot, and mouth disease (HFMD), acute flaccid paralysis (AFP), meningitis, poliomyelitis, and heart disease [1,9]. For example, in EV-A, CV-A2 causes herpangina, EV-A71 causes HFMD and meningitis, and EV-A89 causes AFP [1]. In 1999, a molecular typing method based on alignment of RT–PCR-amplified sequences of the enterovirus *VP1* coding region and *VP1* sequence data of all serotypes (the nucleotide sequence consistency of the same strain *VP1* region was greater than 75%) was used to determine the enterovirus serotype [10]. Molecular typing based on the *VP1* coding region has gradually replaced the conventional neutralization test [11]. This method can be used to classify new enteroviruses that cannot be classified by traditional neutralization tests [12].

To date, EV-A includes 25 serotypes: CV-A2-A8, CV-A10, CV-A12, CV-A14, CV-A16, EV-A71, EV-A76, EV-A89–A92, EV-A114, and EV-A119–A125. EV-A120 is a recently identified serotype of EV-A, and its prototype strain (*LK021688/MAD-2741-11/2011*) was isolated from the faeces of a healthy three-year-old child living in Madagascar in 2011. Later, only a few other EV-A120 strains were isolated in Papua New Guinea, Nigeria, France, Pakistan, Tajikistan and Africa. In Papua New Guinea and Nigeria, the EV-A120 strains were isolated from patients with AFP; In Africa, France and Pakistan, the EV-A120 strains were isolated from wastewater [13–15]. Only one full-length genome sequence (the EV-A120 prototype strain) is currently available in GenBank along with ten EV-A120 partial sequences (five from Africa, and one each from Nigeria, France, Pakistan, Tajikistan, and Papua New Guinea). However, there has been no report about the EV-A120 sequence from China. In this study, we report the full-length genomic sequence of the EV-A120 strain and its genetic characteristics (*Q0082/XZ/CHN/2000*, later defined as *Q0082*). This strain was isolated from the stool of a two-year-old boy with AFP in Tibet in 2000. This is the first report of EV-A120 in China describing its phylogeny, recombination, and epidemiological characteristics.

## Materials and methods

### Sample collection

This study involved no human experimentation. Stool sample of an AFP patient and 48 serum samples from healthy children in Tibet served as the experimental specimens. The research material was a stool sample collected from a 2-year-old boy with AFP in Tibet, China, for public health purposes. Further, the child's parents agreed to the use of his stool samples for research purposes and to the collection of samples by trained professional staff. This study was supported by the Second Ethics Review Committee of the National Institute for Viral Disease Control and Prevention, Chinese Center for Disease Control and Prevention. All experimental protocols were approved by the National Institute for Viral Disease Prevention and Control, and all methods were carried out in accordance with the approved guidelines.

In the serum neutralization experiment, 48 serum samples were collected from 48 healthy children under 5 years of age, including 24 from Lhasa area and 24 from Shigatse area. The Tibet autonomous region Center for Disease Control and Prevention obtained informed consent from all the children's parents during the serum collection process; none of the children showed any disease characteristics at the time of serum collection.

### Virus isolation, purification, and molecular typing

The stool sample collected from the child was treated using the standard procedure, and cultured in human rhabdomyosarcoma (RD) cells and human laryngeal epidermoid carcinoma (HEp-2) cell lines for virus isolation [16]. These two cell lines were provided by the WHO Global Poliovirus Specialized Laboratory in the United States and were originally purchased from the American Type Culture Collection (Manassas, Virginia, USA). Only RD cells showed enterovirus-like cytopathic effect (CPE). After complete enterovirus-like CPE appeared, we collected the infected cell culture for plaque purification. The harvested culture was diluted with 4 gradients (10^−2^, 10^−3^, 10^−4^, 10^−5^), and the diluted cultures were inoculated into a 6-well culture plate containing RD cells. After adsorption at 35°C for 2 hours, agar medium without neutral red solution was added, and culture was inverted at 37°C for 2 days. Then, agar medium containing neutral red solution was added, and cultured in the dark (35°C). After staining, white spots were observed. The appropriate plaques were selected and inoculated into RD cell culture tubes[17,18]. After the appearance of CPE, the viruses were passaged twice, and the cultures were collected.

Viral RNA was extracted from cell cultures using the QIAamp Viral RNA Mini Kit (Qiagen, Hilden, Germany). Then, the One Step PrimeScript™ RT–PCR Kit (Perfect Real Time, TaKaRa, Dalian) was used to detect viral RNA by enterovirus-specific real-time polymerase chain reaction (real-time PCR) [19]. If the real-time PCR results were positive, the PrimeScript One Step RT–PCR Kit Ver.2 (TaKaRa, Dalian, China) and primers E486/E488 were used to amplify the *VP1* region [20]. The PCR product was purified using a QIAquick PCR purification kit (Qiagen, Hilden, Germany), and sequenced in both directions using an ABI 3130 Genetic Analyzer (Applied Biosystems, Foster City, CA, USA), with each strand sequenced at least once. The EV Genotyping Tool and BLAST server were used to identify the enterovirus type, based on the obtained partial *VP1* sequences [21].

### Full-length genome sequencing and partitioning

The full-length genome sequence of the *Q0082* strain was amplified through the “primer-walking” strategy, to close the gaps as necessary [4]. Overlapping segments representing the entire genome were amplified by RT–PCR using specific primers (Table 1). The RT–PCR products were purified using a QIAquick PCR Purification Kit (Qiagen, Hilden, Germany) and sequenced on an ABI 3130 Genetic Analyzer (Applied Biosystems, Foster City, CA, USA) as described above; each nucleic acid site was sequenced at least twice to ensure accuracy. The 5′ end of the genome was amplified using the 5′-Full RACE Kit (Takara, Shiga. Japan) according to the manufacturer's instructions. The 3′ end of the genome was amplified using an oligo-dT primer (7500 A) previously reported in another study [22].

**Table 1.** RT-PCR and sequencing primers.

Primer	Nucleotide Position (nt)	Primer sequence (5′-3′)	Orientation	Reference
0001S48		GGGGACAAGTTTGTACAAAAAAGCAGGCTTTAAAACAGCTCTGGGGTT	Forward	[22]
852A	852–872	GCTGAGGCAGCATAGGAATC	Reverse	This study
EVP4	541–560	CTACTTTGGGTGTCCGTGTT	Forward	[23]
0L68-1	1178–1197	GGTAAYTTCCACCACCANCC	Reverse	[23]
626S	626–649	GCTATTGGATTGGCCATCCGGTG	Forward	This study
1539A	1539–1559	ACTGTGTTAATATATGGCAC	Reverse	This study
1370S	1370–1391	CACAGGAGTAGAGCACACGCA	Forward	This study
2249A	2249–2270	TTACCGGTGGCTTGTGTCCTG	Reverse	This study
E486	2297–2322	TGGTAICARACIAAITWYGTIGTNCC	Forward	[20]
E488	3063–3038	GTIGGRTAICCITCITARAACCAYTG	Reverse	[20]
3216S	3216–3237	GAGGGTGGATACCCAGGCCCA	Forward	This study
4664A	4664–4684	GGGATTTTGACACAGGTCGT	Reverse	This study
4119S	4119–4140	GCAAAGGGGCTGGAATGGATC	Forward	This study
4989A	4989–5010	TGACCAGTTCTGACACCACAG	Reverse	This study
4779S	4779–4799	TCTGATGCCATCCGCCGCAG	Forward	This study
5578A	5578–5598	AGGTTGACACCTTGCTCGTC	Reverse	This study
5394S	5394–5417	GAGCCTTGATTTTGCTCTGTCCC	Forward	This study
6382A	6382–6402	GCAAGTCCAGGCCGTATTTA	Reverse	This study
6039S	6039–6061	GAGGGCAATAAGGAACCAGCGG	Forward	This study
6742A	6742–6762	CACATGGTGGGTGTGGTTTA	Reverse	This study
6569S	6569–6593	GTGTAACCCAGACGTGTTCTGGAG	Forward	This study
7500A		GGGGACCACTTTGTACAAGAAAGCTGGG(T)_24_	Reverse	[22]

As the information of genome division is not available for the EV-A120 prototype strain in GenBank, genome division of the Tibet EV-A120 strain was conducted in this study. The whole genome sequences of EV-A prototype strains (except the EV-A120 prototype strain) and EV-A120 strain (*Q0082*) were compared using MUSCLE software (version3.8.31_i86linux32), and then MEGA (version7.0) was used to construct a maximum likelihood (ML) phylogenetic tree [24], with 1000 bootstrap replicates. ModelGenerator0.85 was used to process and calculate the sequence nucleotide substitution model, and GTR + G was determined as the optimal nucleotide substitution model. Simultaneously, RAxML software (version8.2.12) was used to verify the tree topology [25]. Nucleotide sequence similarity was analyzed using BioEdit (version7.0.9.0) [26]. Finally, CV-A14 (78.1%), CV-A6 (77.8%), CV-A4 (76.0%), and EV-A114 (75.5%), which were close to the EV-A120 strain (*Q0082*) on the ML tree and had high nucleotide similarity, were selected to partition the EV-A120 strain (*Q0082*). These four prototype strains in GenBank were all partitioned. Some basic principles for translating codons into amino acids (including three codons into one amino acid and termination codon characteristics) were incorporated in the partitioning. Further, the EV-A120 prototype strain was partitioned according to the partition characteristics of the Tibet EV-A120 strain.

### Phylogenetic and reorganization analysis

Full-length genomic sequences of the EV-A120 strain and deduced amino acid sequences were aligned with the EV-A prototype strains using the MUSCLE algorithm implemented in MEGA (version7.0). A nucleotide identity matrix and amino acid identity matrix were generated using BioEdit (version7.0.9.0). ML trees were constructed using the GTR + G model as suggested by ModelGenerator0.85 and were implemented in MEGA with 1000 bootstrap replicates. Maximum likelihood trees were also constructed using RAxML (version8.2.12) to verify the best topology. BLAST server was used to analyse the *P2* and *P3* coding region sequences of this strain and to compare them with the sequences in GenBank. Sequences with more than 85% and 85.5% similarity to the *P2* and *P3* regions of the EV-A120 strain (*Q0082*), respectively, were identified as potential parents of the strain and were downloaded from GenBank. The Recombinant Detection Program (RDP, version4.46) was used to screen for recombination signals using seven methods (RDP, GENECONV, MaxChi, Bootscan, Chimaera, SiScan, and 3Seq) [27]. Phylogenetic incongruence between different regions with *P* values less than 0.05 was considered strong evidence for recombination. We only considered recombination events that were identified by at least three methods. To better observe the recombination phenomenon, the serotypes were divided into four groups according to the obtained sequence. Five CV-A2 sequences form the CV-A2 group (blue line); five CV-A4 sequences form the CV-A4 group (grey line); seventeen CV-A6 sequences form the CV-A6 group (green line); one CV-A16 sequence forms the CV-A16 group (brown line). SimPlot (version 3.5.1) was used for similarity plots and bootscanning analysis, with a 200-nucleotide window moving in 20-nucleotide steps [28].

### Neutralizing test

After viral culture purification by the plaque assay, the viral titre of the EV-A120 (*Q0082*) was measured three times and the average value was obtained, followed by determining the serum antibody titres by neutralization test [29]. In total, 48 serum samples were inactivated at 56°C for 30 minutes, and then diluted with five gradients from 1:4 to 1:1024 (1:4, 1:16, 1:64, 1:256, 1:1024) for detection. Next, 50 μL of each serum dilution was added to the 96-well plate and 50 μL of virus was added to each well, which confirmed that each well was 100 CCID_50_. Then the 96-well plate was placed in a 5% CO_2_ incubator at 36°C for 2 hours. Two wells in each column were used as serum controls. We also used a new plate for a cell control and virus control that we called a virus back-titration. Next, 100 μL of RD cells was added to each well and incubated at 36°C with 5% CO_2_ for 7 days, with intermittent observation [30,31]. Only when the virus titre in the back-titration plate was between 32 and 320 CCID_50_ and when the cell control was acceptable did we judge the result as effective. The maximum dilution of serum protecting 50% of the culture at the end of 7 days was recorded based on the EV-like cytopathic effect. If the titre of the observed neutralizing antibody was greater than the 1:8 dilution, the serum sample was considered positive and the geometric mean titre (GMT) was calculated accordingly [32,33]. We used SPSS Statistics software (version19.0) (SPSS Inc., Chicago, IL, USA) for statistical analysis and performed the chi-square test to compare the differences in seroprevalence between Lhasa and Shigatse regions.

### Assay for temperature sensitivity

The plaque-purified EV-A120 strain (*Q0082*) and two selected control strains (*HTYT-ARL-AFP02F/XJ/CHN/2011*, showing no temperature sensitivity and *HTYPS-QDH11F/XJ/CHN/2011*, showing temperature sensitivity) were examined for temperature sensitivity on monolayer RD cells in 24-well plates [34–36]. The 24-well plates were inoculated with 50 μL of undiluted virus stocks. Two different incubation plates were used: one was placed at the optimum temperature of 36°C, and the other was placed at the super-optimal temperature of 39.5°C for virus propagation. After adsorption at 36°C or 39.5°C for 1 hour, non-adsorbed virus cultures were discarded and 100 μL of maintenance fluid was added to each well. The 24-well plates were then incubated at 36°C and 39.5°C, respectively, and sampled at five time points after infection (4, 8, 16, 24, and 48 hours). CCID_50_ was calculated by the end-point dilution of monolayer RD cells in a 96-well plate at 36°C. Virus isolates that show a drop of more than 2 log values at different temperatures were considered temperature sensitive [29,34,36,37].

## Results

### Virus isolation and molecular typing

**Figure 1.**
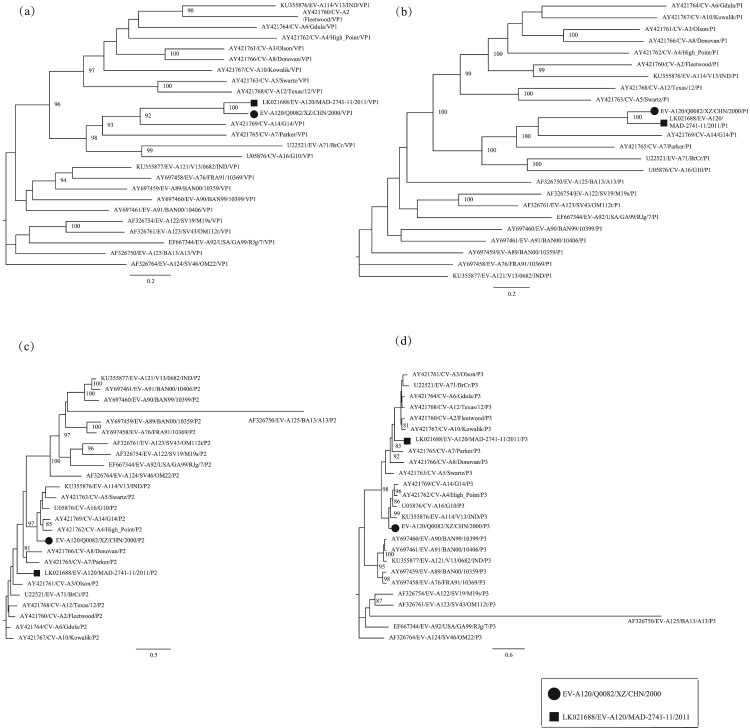
Phylogenetic tree based on the *VP1*, *P1*, *P2,* and *P3* sequences of EV-A. Maximum likelihood trees were constructed using the GTR + G model and were implemented in MEGA7.0 with 1000 bootstrap replicates. The circle and square represent the *Q0082* strain and the EV-A120 prototype, respectively. Scale bars indicate the genetic distance. All panels have the same proportions. (a) *VP1* coding sequence; (b) *P1* coding sequence; (c) *P2* coding sequence; (d) *P3* coding sequence.

One of the main characteristics of enteroviruses is cytolytic effects in cell culture [1]. The EV-A120 prototype strain induces CPE in both RD cells and HEp-2 cells [13], whereas the *Q0082* strain only showed CPE in RD cells. After the entire *VP1* region sequences were amplified, analytical results of the Online Enterovirus Genotyping Tool and BLAST showed that this strain belonged to the EV-A120 serotype . The phylogenetic tree based on the *VP1* coding region also verified that this strain was EV-A120 (Figure 1a).

### Gene partition of the Q0082 strain

**Figure 2.**
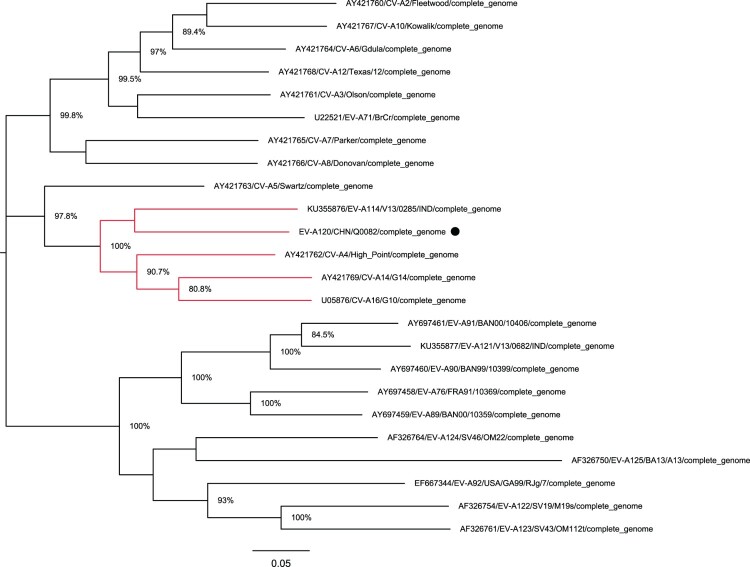
Phylogenetic tree based on the full-length genomic sequences of EV-A. Nucleotide sequences of the *Q0082* strain (represented by circles) and 23 other EV-A prototype strains were compared using MUSCLE software. A maximum likelihood phylogenetic tree was constructed using MEGA7.0. The branches marked in red represent the prototype strains clustered with the Tibet EV-A120 strain.

The full-length genomic sequence of the *Q0082* strain was determined. However, there was no information on the genomic partition of EV-A120 in GenBank. According to a phylogenetic tree based on its full-length genome, the *Q0082* strain was found to be clustered with EV-A114 (*KU355876/ v13-0285 /IND/2013*), CV-A14 (*AY421769/G-14/USA/2003*), CV-A6 (*AY421764/Gdula/USA/2003*), and CV-A4 (*AY421762/High_Piont/USA/2003*) (Figure 2). The nucleotide sequences and deduced amino acid sequences of this isolate were compared with other EV-A prototype strains. Then, according to the gene partition of EV-A114 prototype strain and other EV-A prototype strains, the *Q0082* strain was also partitioned. Results showed that the sequence length of the *5′-UTR* region was 749 nt, that of *P1* region was 2580 nt, of *P2* region was 1734 nt, of *P3* region was 2259 nt, and that of the *3′-UTR* region was 84 nt.

### Full-length genomic characterization of the Q0082 strain

The genome of the *Q0082* strain was 7406 nt long. The length of the open reading frame (ORF) was 6576 nucleotides, which encodes 2192 amino acids. The *5′-UTR* region had 749 nt and the *3′-UTR* region had 84 nt. Compared with the EV-A120 prototype, the *Q0082* strain showed two nucleotide deletions at nucleotide 101 and 7337, and three nucleotide insertions at nucleotide 695. The base compositions of the Tibet EV-A120 strain was 27.4% A, 24.8% U, 24.2% C, and 23.6% G.

Pairwise comparison of nucleotide sequences and deduced amino acid sequences was performed among the *Q0082* strain and the EV-A120 prototype and other EV-A prototype strains. The full-length genome sequence of the *Q0082* strain and the EV-A120 prototype strain revealed 79.9% similarity in nucleotide sequence and 96.3% similarity in amino acid sequence. The nucleotide sequence similarity and amino acid sequence similarity of the *P1* region sequence of *Q0082* strain and EV-A120 prototype strain was 81.3% and 96.3%, respectively. The nucleotide sequence similarity compared with other EV-A prototype strains was 61.6%–69.1%, and the amino acid sequence similarity was 66.0%–79.3%. In the *P2* and *P3* regions, the *Q0082* strain shares a distinct high identity (81.3%–87.8%) with other EV-A prototype strains (Table 2). The results suggest that different EV-A120 strains may have undergone recombination in the *P2* and *P3* coding regions.

**Table 2.** Pairwise comparisons of nucleotide sequences and deduced amino acid sequences were performed among the *Q0082* strains, the EV-A120 prototype strain (*LK021688/MAD-2741-11/2011*), and other EV-A prototype strains.

Region	% nucleotide identity (% amino acid identity)			
	Identity with the EV-A120 prototype strain (%)		Identity with other EV-A prototype strains (%)	
	nucleotide	Amino acid	nucleotide	Amino acid
*5′-UTR*	83.5		64.4–88.9	
*VP4*	75.8	91.3	60.8–80.1	66.6–95.6
*VP2*	80.3	98.8	63.3–70.6	63.8–76.9
*VP3*	82.3	97.9	61.8–70.3	66.1–79.8
*VP1*	82.4	94.9	55.4–64.8	55.8–72.7
*2A*	79.3	96.6	62.4–81.3	61.3–96.6
*2B*	74.7	91.9	56.9–84.8	51.5–98.9
*2C*	80.7	98.4	64.6–85.3	66.2–99.3
*3A*	82.1	96.5	55.5–85.6	60.9–98.8
*3B*	77.2	95.4	53.0–87.8	54.5–95.4
*3C*	78.3	96.7	59.3–83.9	59.5–98.3
*3D*	78.5	94.8	62.9–85.0	66.4–97.8
*3’-UTR*	76.6		32.0–89.2	

### Phylogenetic analysis of the Q0082 strain

Both partial and entire *VP1* nucleotide sequence analysis can be used to investigate the phylogenetic relationship among human enteroviruses, but entire *VP1* sequence analysis can provide more accurate information [38]. Phylogenetic trees were established based on nucleotide sequences of *VP1*, *P1*, *P2,* and *P3* coding regions of the *Q0082* strain and all EV-A prototype strains in GenBank database (Figure 1). A phylogenetic tree based on *VP1* region showed that this strain clustered with the EV-A120 prototype strain, confirming the previous molecular typing results. A phylogenetic tree based on the *P1* region also showed that this strain was clustered with the prototype strain EV-A120.

However, in the phylogenetic trees based on *P2* and *P3* coding regions, the *Q0082* strain did not converge with the EV-A120 prototype strain. In the *P2* region, the *Q0082* strain clustered with CV-A14 (GenBank no. AY421769), CV-A4 (GenBank no. AY421762), EV-A114 (GenBank no. KU355876), CV-A5 (GenBank no. AY421763), and CV-A16 (GenBank no. U05876). In the *P3* region, the *Q0082* strain clustered with CV-A16, CV-A14, CV-A4, and EV-A114. This suggests that the *Q0082* strain may recombine with other EV serotypes.

### Recombination of the Q0082 strain with other EV-A strains

**Figure 3.**
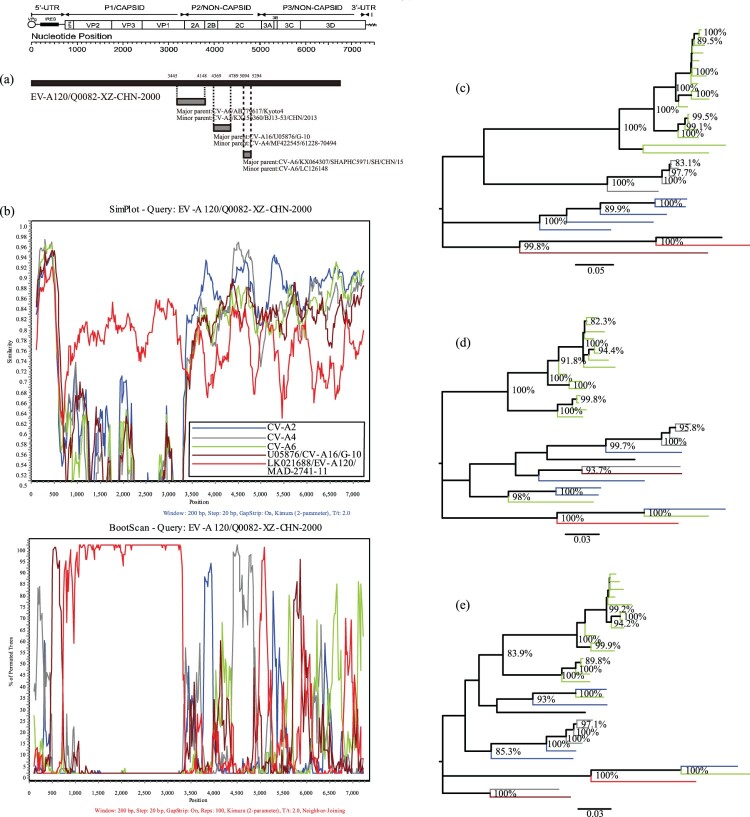
Recombination analyses of the Tibet EV-A120 strain with other EV-A strains (a) Recombination events predicted for the *Q0082* strains. The genome of the Tibet EV-A120 is shown as a black block. Genetic components identified by RDP4 that were involved in recombination events are shown as light grey blocks. Likely breakpoint positions are shown above the genome. (b) Similarity plots and boot scanning analyses of EV-A120, CV-A2, CV-A4, and CV-A6 strains with potential parents. The *Q0082* Strain (*EV-A120/Q0082-XZ-CHN-2000*) was used as a query sequence; (c) Phylogenetic tree of sequences based on the *P1* coding region; (d) Phylogenetic tree of sequences based on the *P2* coding region; (e) Phylogenetic tree of sequences based on the *P3* coding region. All branches of the trees are coloured according to the results of boot scanning analysis. The sequences analyzed in the recombination and phylogenetic trees were identical.

Recombination analysis demonstrated an obvious intraspecies recombination phenomenon. BLAST analysis of the *P2* and *P3* regions of the *Q0082* strain revealed that three serotypes, CV-A2, CV-A4, and CV-A6, were highly similar to *Q0082*. BLAST results combined with the phylogenetic trees based on P2 and P3 regions showed that CV-A2, CV-A4, CV-A6, and CV-A16 were identified as the major and minor putative parents using RDP4 (Version4.46) with seven supported methods (Figure 3a). Using the *Q0082* strain as the query sequence, SimPlot (Version3.5.1) was used to determine the recombination site. The SimPlot results showed that the *Q0082* strain had the highest similarity with the EV-A120 prototype strain in the *P1* region. And sequences of the *Q0082* strain and CV-A4 were recombined in the *5′-UTR* region and *2C* region. In the *5′-UTR* region and 3C region, the *Q0082* strain was recombined with CV-A16. In the *2B* region, recombination was found between the *Q0082* strain and CV-A2 group. In the *3D* region, recombination was observed between the *Q0082* strain and the CV-A6 group (Figure 3b). The phylogenetic tree established with the aforementioned sequences is consistent with the results of SimPlot and BootScanning, which verifies the results of the recombination analysis (Figure 3c, d, e).

### Seroprevalence of the Q0082 strain

In total, 48 serum samples were collected from children in Tibet, including 24 from Lhasa and 24 from Shigatse. Of these, 5 samples were seropositive (>1:8), with a total seropositive rate of 10.42% and a GMT of 1:5.26. The composition ratios for the EV-A120 neutralization antibody titres of <1:8, 1:8–1:64, and >1:64 were 85.42%, 12.5%, and 2.08%, respectively (Table 3). Compared with the serum epidemiological studies of other enteroviruses (such as EV-A71, CV-A16, EV-A89 and EV-A90) in China, the positive rates of EV-A120 neutralizing antibodies and GMTs were significantly lower [2,30,32].

**Table 3.** The EV-A120 neutralization antibody titres.

Titres	Lhasa City		Shigatse area		Total (%)
	Number of samples	Ratio (%)	Number of samples	Ratio (%)	
<1:8	22	91.7	19	79.2	41(85.4)
1:8–1:64	1	4.15	5	20.8	6(12.5)
>1:64	1	4.15	0	0	1(2.1)
Total	24		24		48

In Lhasa, the positive rate of neutralization antibody and GMTs was 8.33% and 1:5.04, respectively, and that in Shigatse region was 12.5% and 1:5.50, respectively. However, chi-square analysis showed no significant difference in the positive rate of neutralization antibody and GMTs between the two cities (positive rate: *P* > 0.05, GMTs: *P* > 0.05).

### Temperature sensitivity of the Q0082 strain

**Figure 4.**
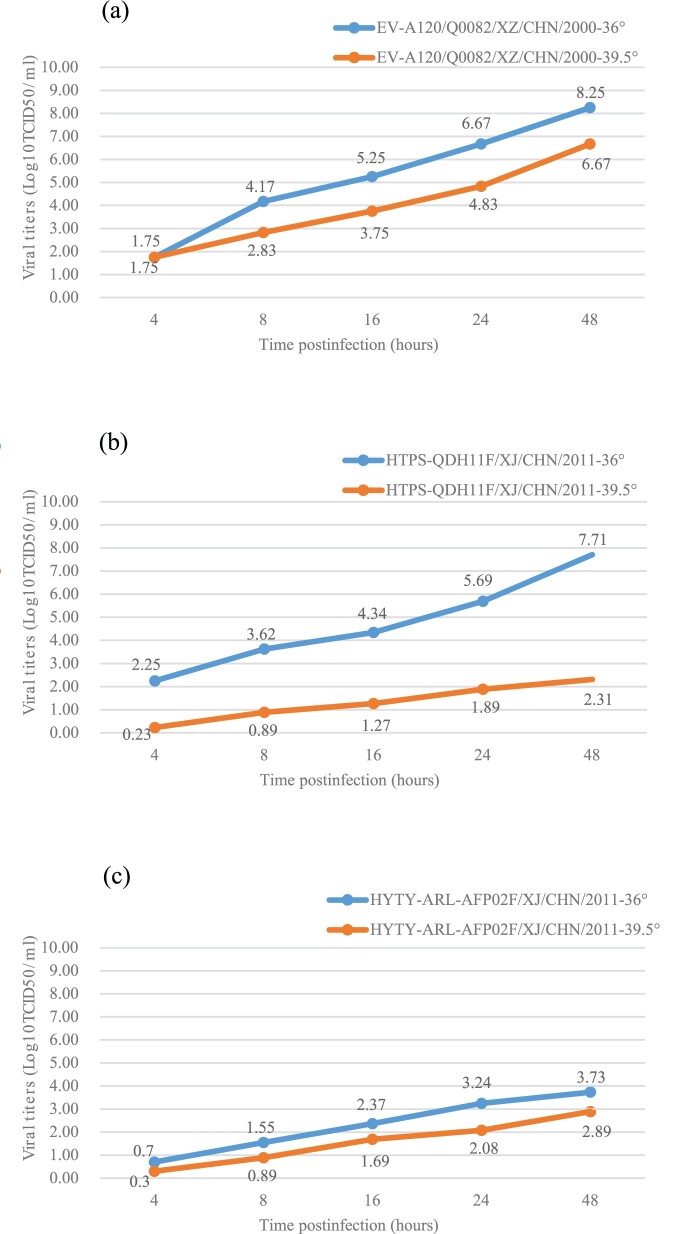
Temperature sensitivity test curves for the EV-A120 strain. Blue and red lines represent the growth trends of the viruses on RD cells at 36°C and 39.5°C, respectively. The Xinjiang EV-B85 strain (*HTYT-ARL-AFP02F/XJ/CHN/2011*, showing no temperature sensitivity) and the EV-B106 strain (*HTYPS-QDH11F/XJ/CHN/2011*, showing temperature sensitivity) were used as experimental controls. (a) Strain *EV-A120/Q0082-XZ-CHN-2000*; (b) Strain *HTYT-ARL-AFP02F/XJ/CHN/2011*; (c) Strain *HTYPS-QDH11F/XJ/CHN/2011*.

Temperature sensitivity usually serves as an in vitro marker for the attenuation of poliovirus vaccine strains and EV-A71, although the link between temperature sensitivity and attenuation may not be straight forward, it could serve as an indicator of virulence in enterovirus[34,37]. Titres of the *Q0082* strains cultured at 36°C and 39.5°C showed that the strain was not temperature-sensitive because the titre reduction was less than two logarithms at 36°C and 39.5°C (Figure 4). Compared with the temperature sensitive EV-A106 strain (*HTYPS-QDH11F/XJ/CHN/2011*) (Figure 4c), the *Q0082* strain showed stronger resistance to temperature.

## Discussion

Neutralization test has long been regarded as an important means of enterovirus typing [39]. However, traditional neutralization experiments require a large amount of laboratory labour and time. Thus, in the event of an outbreak, this method of identifying enterovirus serotypes is impractical. In the early stage of enterovirus infection, rapid and correct diagnosis is necessary. Therefore, “*VP1* region molecular typing” has gradually replaced the neutralization test to become the standard serotyping method for enteroviruses in the laboratory. Using this method, increasing number of new enterovirus serotypes have been identified [38]. EV-A120 is a newly defined enterovirus serotype in 2011 with very few sequences in GenBank: only one full-length genomic sequence and ten partial sequences [40]. Because of the limited literature and the lack of sequence information, studies on EV-A120 can enrich our limited knowledge.

Phylogenetic tree analysis showed that the *Q0082* strain did not converge with the EV-A120 prototype strain in the *P2* and *P3* regions, suggesting the possibility of recombination. Many studies have shown that recombination events occur frequently in enteroviruses [41]. Frequent recombination and mutations in enteroviruses have been recognized as the main mechanisms for their evolution, enabling them to rapidly respond and adapt to new environments; further, it is been reported that *P3* is the most frequently recombined region of enteroviruses [6,40]. The accumulation of interspecies and intraspecies recombination events could be a strong driver of the emergence and disappearance of certain enterovirus serotypes[6,42]. Although recombination between EV-A120 and CV-A2, CV-A4, CV-A6, CV-A16 was possible, it is hardly to ascertain that the CV-A2, CV-A4, CV-A6, or CV-A16 strain was exact the donor for the Tibet EV-A120 strains. However, we can speculate that Tibet EV-A120 strains and CV-A2, CV-A4, CV-A6, CV-A16 strains might have co-circulated with other EV-A and have had extensive genetic exchanges with other EV-A strains, thus, it is likely that the related P3 sequences of Tibet EV-A120 originated through a common unknown EV-A ancestor through recombination. Based on BLAST and RDP analysis, CV-A2, CV-A4, CV-A6, and CV-A16 were identified as the major and minor putative parents. To verify recombination and identify the recombination sites, SimPlot and BootScan analyses were conducted, which revealed recombination between the *Q0082* strain and some sequences of CV-A2, CV-A4, CV-A6, and CV-A16. Notably, recombination analysis of the *Q0082* strain showed partial recombination with CV-A4 and CV-A16 in the *5′-UTR* IRES region. The *5′-UTR* of enterovirus has a clover-like secondary structure, and IRES play an important role in RNA replication and viral protein translation [43,44]. Despite insufficient evidence, we hypothesized that recombination in the *5′-UTR* region may have affected viral replication, leading to different growth abilities in different cells.

To investigate the prevalence of the *Q0082* strain in Tibet, China, a serum neutralization experiment was conducted. The results of low serum-positive rate and GMTs showed that the extent of EV-A120 transmission and exposure of the population in China was much lower than that of EV-A71 and CV-A16 since 2008 [32]. Moreover, there was no significant difference in the neutralization antibody positive rate and GMTs in the Lhasa and Shigatse areas of Tibet, China (*χ*^2^ = 0.416, *P* > 0.05), suggesting that there was no significant geographical difference in the prevalence of the *Q0082* strain. Although the *Q0082* strain is not sensitive to the temperature, it has not caused epidemics in the population according to the results of serum neutralization test, and no disease outbreaks caused by EV-A120 have been reported before.

To determine that an enterovirus is the causive pathogen of the disease, it needs the support of strong epidemiology, clinical medicine and laboratory evidence. And it also needs to be tested at the animal level to determine whether the pathogen can cause the similar disease. In all studies including this study in the world, EV-A120 was isolated from only sporadic patient with AFP, and healthy people. Although this is consistent with the pathogenic characteristics of enteroviruses, there is still insufficient epidemiological and laboratory evidence to prove that EV-A120 is the pathogenic pathogen of AFP. However, EV-A120 as a new emerging pathogen, we are using cell level experiments and animal experiments to verify its pathogenicity, which provided strong evidence for the relationship between EV-A120 and AFP.

In China, the AFP case surveillance system plays an important role in detecting new enteroviruses. The discovery of novel enteroviruses (such as EV-A89, EV-A90, EV-B80, EV-B85, EV-B106, and EV-C96) provides important information for studying the molecular epidemiological characteristics of these novel enteroviruses. Because of the AFP case surveillance system, the *Q0082* strain was isolated. However, a detailed study of EV-A120 is difficult as only this strain of EV-A120 was isolated in China, only few EV-A120 strains are reported in the world, and limited epidemiological data about EV-A120 are available. Overall, this is the first report describing the detailed genome and some biological characteristics of the new enterovirus EV-A120 from China. The low isolation rate of EV-A120 indicates that it is not a prevalent enterovirus serotype in China or worldwide. However, because it is a new pathogen, the vast majority of people do not have immunity, and hence, detailed studies are necessary to prevent possible outbreaks [45]. The results of serum neutralization tests and temperature sensitivity also clarified the genomic structure and epidemiological information of EV-A120, which can play some role in the control of enterovirus-related diseases. As the isolation rate of EV-A120 is relatively low, we have been unable to perform detailed study on its biological and pathological characteristics. However, we believe that this study provides a solid foundation for further studies on EV-A120, and expands the full-length genome sequence information in GenBank.

## Data Availability

Full-length genome nucleotide sequences for the *Q0082* strains determined in this study have been deposited to the GenBank nucleotide sequence database under accession number MT123346.
